# Genomic Analysis of *Pseudomonas aeruginosa* Recovered From Surgical Site Infections From a Referral Hospital in Western Kenya Reveals Dominance of High‐Risk Subtypes

**DOI:** 10.1155/ijm/3640494

**Published:** 2026-08-30

**Authors:** Sarah Kindiki, Sabella Kiprono, Oleg Reva, Peter Kuloba Nyongesa, Nyabera Nicholas Mogoi, Martin Welch, Anthony Sifuna

**Affiliations:** ^1^ Department of Medical Laboratory Sciences, Masinde Muliro University of Science and Technology, Kakamega, Kenya, mmust.ac.ke; ^2^ Department of Biochemistry, Genetics and Microbiology, Centre for Bioinformatics and Computational Biology, University of Pretoria, Pretoria, South Africa, up.ac.za; ^3^ Department of Medical Microbiology and Parasitology, Masinde Muliro University of Science and Technology, Kakamega, Kenya, mmust.ac.ke; ^4^ Centre for Epidemic Response and Innovation, School for Data Science and Computational Thinking, Stellenbosch University, Stellenbosch, South Africa, sun.ac.za; ^5^ Department of Biochemistry, University of Cambridge, Cambridge, UK, cam.ac.uk; ^6^ Department of Medical Biochemistry, Masinde Muliro University of Science and Technology, Kakamega, Kenya, mmust.ac.ke

**Keywords:** genome diversity, multidrug resistance, pathogen evolution, *Pseudomonas aeruginosa*

## Abstract

*Pseudomonas aeruginosa* is an important cause of surgical site infections (SSIs) and is characterized by extensive antimicrobial resistance and genomic plasticity. We analyzed 13 whole‐genome sequences of clinical *P. aeruginosa* isolates recovered from SSIs at a Level 5 referral hospital in Western Kenya and integrated genomic analyses with antimicrobial susceptibility tests and phenotyping of quorum‐sensing (QS). Multidrug resistance was common, with resistance observed primarily against ciprofloxacin, piperacillin, ceftazidime, and amikacin, whereas meropenem and piperacillin/tazobactam retained the greatest in vitro activity. Genomic analyses identified diverse sequence types, extensive variability in insertion sequences and genomic islands, and widespread conservation of intrinsic resistance determinants, whereas accessory antimicrobial resistance genes were infrequently detected and largely associated with genomic islands. Virulence‐associated genes were predominantly chromosomal, although several showed strain‐specific genomic island localizations. Functional QS analyses demonstrated that isolates with intact *lasR* and *rhlR* produced significantly higher levels of acyl‐homoserine lactone signals than isolates carrying predicted loss‐of‐function mutations, whereas Pseudomonas quinolone signal production remained comparatively conserved. These findings provide a genomic baseline for *P. aeruginosa* causing SSIs in Western Kenya and highlight the importance of integrating whole‐genome sequencing with phenotypic analyses to strengthen surveillance and inform antimicrobial stewardship and infection prevention strategies.

## 1. Introduction

Surgical site infections (SSIs) remain a significant burden in healthcare settings, contributing to prolonged hospital stays, increased healthcare costs, and elevated morbidity and mortality rates [1, 2]. In sub‐Saharan Africa, the opportunistic gram‐negative human pathogen, *Pseudomonas aeruginosa,* is implicated in many SSIs [3]. *P. aeruginosa* is a formidable pathogen due to its intrinsic resistance to many antibiotics and its ability to acquire additional resistance determinants via mutations and horizontal gene transfers [4–7]. It is commonly associated with many hospital‐acquired infections, and thus poses a severe challenge to infection control, particularly in resource‐limited settings.

Clinical isolates of *P. aeruginosa* have been intensively studied from infection sources in the developed world for many years now, but we know much less about the pathogen in low‐ and middle‐income (LMIC) settings such as sub‐Saharan Africa. Despite the growing global application of whole‐genome sequencing to characterize clinical *P. aeruginosa* populations [8], comparable genomic data from SSIs in sub‐Saharan Africa remain scarce. In particular, little is known about the distribution of high‐risk sequence types, the contribution of horizontally acquired genomic islands (GIs) to antimicrobial resistance (AMR) and virulence [9]. Additionally, there is a dearth if information on the relationship between these acquired determinants and quorum‐sensing (QS) regulatory pathways in isolates circulating within the region. Addressing these knowledge gaps is important for understanding the evolution and clinical behavior of locally circulating strains. Here, and to investigate this further, we characterized a selection of *P. aeruginosa* isolates recovered from patients with SSIs at a referral health facility (Kakamega County General Hospital [KCGH]) in Western Kenya. *P. aeruginosa* exhibits variable resistance to multiple classes of antibiograms commonly used for the treatment of SSIs. These include *β*‐lactams, fluoroquinolones, aminoglycosides, and carbapenems. This increasing resistance complicates treatment and underscores the need for continued surveillance of both resistance and virulence determinants. Given that the management and treatment of SSIs may be severely compromised by the presence of AMR determinants, and by the ability of the organism to acquire resistance and virulence determinants through horizontal gene transfer, particular attention was paid to the complement of AMR‐associated genes and their location (on the core chromosome or on horizontally acquired islands). The presence of horizontally acquired virulence determinants was intriguing because, in laboratory strains of *P. aeruginosa*, numerous virulence‐associated traits, including biofilm formation, motility, and secretion of extracellular toxins, are regulated by QS [10, 11]. Previous studies have also demonstrated that QS largely influences how genes located outside the conserved core genome are expressed, and how it contributes to the integration of newly acquired functions into existing regulatory networks [12, 13]. We see these observations raising the possibility that virulence factors encoded on horizontally acquired GIs may likewise become incorporated into QS‐controlled pathways. As a step towards investigating this possibility, we examined the genetic integrity of known QS regulators in these strains and assessed their ability to synthesize QS signal molecules. To place this study into context, KCGH receives approximately 15,000 patients monthly from the region and has a bed capacity of 400, so our work likely captures a reasonable snapshot of the *P. aeruginosa* SSI burden in the region.

## 2. Methods

### 2.1. Study Design

KCGH is one of the largest referral hospitals in Western Kenya, with a bed capacity of 400. This study adopted an analytical cross‐sectional design with purposive sampling to select patients likely to have *P. aeruginosa* infections, based on clinical assessments or prior medical history. Samples for the study were selectively collected during the hospitalization period. This cross‐sectional study was not tied to an age group, but rather involved all patients who had consented to participate in this study between January 2022 and October 2022. Specimens and isolates included in this study were collected from the 128 male and female patients, out of 209 screened patients with SSIs from general surgery. Specimens were collected from patients who presented with postoperative SSIs occurring within 1 month of the surgical procedure. Surgical procedures included in this study were exploratory laparotomy, extirpation/resection, incision and drainage, surgical debridement, amputation and other orthopedic surgeries, cesarean section, circumcision, urologic/vascular access procedures, escharotomy/fasciotomy/skin grafting, and flap reconstruction/debridement. Patients with documented prolonged or repeated antimicrobial treatment before surgery, as identified from their clinical records, were excluded. This criterion was applied to minimize the influence of pre‐existing antimicrobial selection pressure on pathogen recovery and susceptibility profiles associated with the SSI. Among the 128 samples collected, only 13 *P. aeruginosa* samples were selected for the analysis of this work as described in Section 2.2.3.

Ethical approval for this study was sought from the Institutional Scientific and Ethics Review Committee (ISERC) of MMUST (Approval No. MMUST/IERC/176/2023) and the National Commission for Science, Technology, and Innovation (NACOSTI) License No. NACOSTI/P/23/28712. Authorization for data collection was obtained from the Kakamega County Director of Medical Health Services (No. ERC/226‐11/2023). Moreover, consent procedures were done by the use of a consent form and a data collection questionnaire (File S9).

### 2.2. Data Collection and Laboratory Procedures

#### 2.2.1. Isolation and Recovery of *P. aeruginosa* From Surgical Wounds

Patients admitted to surgical wards were routinely monitored for clinical signs indicative of SSIs, including pain, redness, warmth, swelling, and the presence of purulent discharge at the incision site. SSIs were diagnosed by a trained clinician based on the standardized criteria provided by the US Centers for Disease Control and Prevention (CDC), which classifies SSIs into superficial incisional, deep incisional, and organ/space infections. This classification also provided a basis for examining correlations between the type of SSI and the virulence or AMR profiles of the bacterial isolates recovered.

Initial isolation and identification of *P. aeruginosa* isolates were performed using selective and differential media, including cetrimide agar, MacConkey agar (MAC), and blood agar (BA) (Oxoid, United Kingdom). Inoculated plates were incubated aerobically at 37°C and examined for visible bacterial growth after 24 h. Colonies with characteristic morphology were subjected to Gram staining and a series of biochemical tests, including sulfur, indole, and motility (SIM), triple sugar iron (TSI), urease, citrate, oxidase, and growth at 42°C, following standard microbiological protocols [14]. For long‐term storage, purified single colonies were suspended in tryptic soy broth (TSB) supplemented with 30% glycerol (*v*/*v*) and stored at −20°C.

#### 2.2.2. Antimicrobial Sensitivity Tests

Antimicrobial susceptibility testing was performed for each isolate of *P. aeruginosa*. The Kirby–Bauer disk diffusion technique [15] was used to do this, and the following antibiotic disks were tested: ciprofloxacin (CIP) (5 *μ*g), piperacillin (PIP) (100 *μ*g), piperacillin/tazobactam (TZP) (100/10), amikacin (AK) (30 *μ*g), meropenem (MRP) (10 *μ*g), tobramycin (NN) (10 *μ*g), and ceftazidime (CAZ) (30 *μ*g). These antibiotics were chosen because they represent multiple classes of clinically relevant antibiotics, including the aminoglycosides (AK and NN), an antipseudomonal carbapenem (MRP), a cephalosporin (CAZ), beta‐lactams (TZP and PIP), and an antipseudomonal fluoroquinolone (CIP). Tests were carried out on Mueller–Hinton agar (MHA) (Oxoid, United Kingdom), and the plates were incubated at 37°C for 24 h before recording. The inhibition zone sizes were interpreted as described by the Clinical and Laboratory Standards Institute (CLSI) [16]. Quality control was performed by testing ATCC reference strains (*P. aeruginosa* ATCC27853 and *Escherichia coli* ATCC 25922).

#### 2.2.3. DNA Isolation and Sequencing

Genomic DNA was extracted from 13 selected *P*. *aeruginosa* isolates using the Zymo Research Genomic DNA Extraction Kit (17062 Murphy Ave., Irvine, California, 92614), following the manufacturer′s protocol. The extracted DNA was sequenced using the Illumina MiSeq platform. Raw sequencing reads were subjected to quality control using FastQC to assess base quality, GC content, and adapter contamination. Low‐quality bases and sequencing adapters were removed using Trimmomatic, with parameters set to trim reads with a Phred score below 20 and retain only reads where at least 97% of bases met this quality threshold. Reads that were too short after trimming were excluded from downstream analysis.

#### 2.2.4. Genome Annotation and Comparison

Bowtie2 implemented in Unipro UGENE v44.0 was used for read alignment against the reference genome of *P. aeruginosa* PA01 using default parameters unless otherwise specified. The PAO1 genome was used primarily as a reference framework for comparative analyses and consensus sequence generation. Subsequent genome annotation, orthologs identification, sequence typing, and GI analyses were performed on the assembled consensus genomes, thereby reducing dependence on the reference genome for downstream comparative analyses. Consensus sequences were generated from the read alignments using samtools and annotated using PROKKA v1.14.5 [17] implemented in the Kbase Web‐server (https://www.kbase.us/). PROKKA v1.14.5 was executed using default annotation parameters. The Comprehensive Antibiotic Resistance Database (CARD‐RGI) resistance gene identifier [18] was used to search for antimicrobial‐resistant genes. Additionally, genetic virulence determinants were searched using the reference virulence factor database (VFDB) [19].

The obtained chromosomal sequences of the isolates were used for MLST typing via the PubMLST web portal (https://pubmlst.org/). Phylogenetic relationships among the strains were inferred using protein sequences of 2978 orthologous protein‐coding genes identified by default‐set OrthoFinder [20] in the annotated genomes of the isolates, along with the genome of *Pseudomonas fluorescens* (NC_00412), which was used for tree rooting. Each set of orthologous proteins was aligned using MUSCLE [21] using default parameters. The resulting alignments were concatenated into a superalignment totaling 975,355 amino acid residues. MEGA 12 [22] was used to construct the phylogenetic tree using the Neighbor‐joining (NJ) algorithm, applying the JTT amino acid substitution mode. Pairwise deletion of gaps and missing data was applied during phylogenetic reconstruction. Locations of horizontally acquired GIs in the sequenced genomes were identified using the SeqWord Gene Island Sniffer program [23]. Because the primary objective was comparative genomic characterization relative to a well‐curated *P. aeruginosa* reference genome, a reference‐guided assembly approach was employed. We acknowledge that de novo assembly may recover accessory genomic regions that are absent from the reference genome and therefore represents a potential avenue for future investigation.

#### 2.2.5. QS Assay

Production of the QS signal molecules Pseudomonas quinolone signal (PQS), N‐3‐oxododecanoyl‐L‐homoserine lactone (OdDHL), and N‐butanoyl‐L‐homoserine lactone (BHL) was assessed using established bioluminescent reporter strains as previously described by [24]. The biosensor strains *P*. *aeruginosa* PAO1 *Δ*pqsA CTX‐lux::pqsA (for PQS detection), *E*. *coli* JM109 (pSB1075) (for OdDHL detection), and *E*. *coli* JM109 (pSB536) (for BHL detection) were used. For the bioassays, equal volumes of exponentially growing biosensor culture and test sample were mixed and incubated with shaking at 37°C for 3 h. All assays were performed in triplicate. Bioluminescence was measured by single‐point luminometry using a Lucy 1 luminometer (Anthos Labtec Instruments, Austria). Sterile Luria broth served as the background control, although background luminescence was not subtracted from the recorded measurements. *P. aeruginosa* PA14 and PAO1 were included as high‐ and moderate‐virulence reference strains, respectively, to validate reporter strain responses. For downstream genotype–phenotype analyses, isolates were classified according to predicted QS regulator function as Intact, comprising isolates with intact lasR and rhlR, or loss‐of‐function (LOF), comprising isolates carrying predicted LOF mutations in lasR and/or rhlR. This classification was used to compare QS signal production, determine whether QS regulator genotype predicted the observed QS phenotype (BHL+/OdDHL+/PQS+ vs. PQS‐only), evaluate differences in biofilm biomass quantified by crystal violet staining (OD<sub>570</sub>), and assess associations between accessory AMR gene carriage (presence/absence) and QS regulator genotype.

#### 2.2.6. Data Analyses

Data processing, statistical analyses, and graphical visualizations were performed in R version 4.5.2 [25]. Antimicrobial susceptibility data were retained as continuous inhibition zone diameters (mm) for quantitative analyses and clustering, whereas a parallel categorical dataset containing the corresponding CLSI susceptibility classifications (susceptible, intermediate, or resistant) was generated for descriptive summaries and visualization. Hierarchical clustering of susceptibility profiles was performed using Euclidean distance matrices derived from inhibition zone diameters. Associations among susceptibility measurements were evaluated using Spearman′s rank correlation coefficient. Categorical susceptibility frequencies were summarized as counts and percentages. Heatmaps were generated using the ComplexHeatmap package, whereas summary plots and statistical graphics were produced using ggplot2.

Multidrug resistance (MDR) burden was defined as the number of antibiotics for which an isolate was classified as resistant according to CLSI criteria. Differences in MDR burden between MRP‐resistant and MRP nonresistant isolates were evaluated using both Student′s *t*‐test and the Wilcoxon rank‐sum test. Luminescence values obtained from the BHL, OdDHL, and PQS biosensor assays were compared between QS genotype groups (Intact vs. LOF) using the Wilcoxon rank‐sum test. Rank‐biserial correlation coefficients were calculated as measures of effect size, and *p* values were adjusted for multiple comparisons using the Benjamini–Hochberg false discovery rate (FDR) procedure. Technical triplicates were included for each isolate and are displayed individually in graphical summaries. Associations between QS regulator genotype and QS phenotype were evaluated using Fisher′s exact test, with contingency tables used to calculate odds ratios and corresponding *p* values. Differences in biofilm biomass between QS genotype groups were also assessed using the Wilcoxon rank‐sum test. Associations between accessory AMR gene carriage (presence/absence) and QS regulator genotype were evaluated using Fisher′s exact test because of the small sample size and sparse contingency tables, with *p* values adjusted using the Benjamini–Hochberg FDR procedure. Unless otherwise stated, all statistical tests were two‐tailed, and statistical significance was defined as an adjusted *p* value < 0.05 where multiple‐testing correction was applied, or *p* < 0.05 otherwise.

## 3. Results

### 3.1. AMR

**Figure 1:**
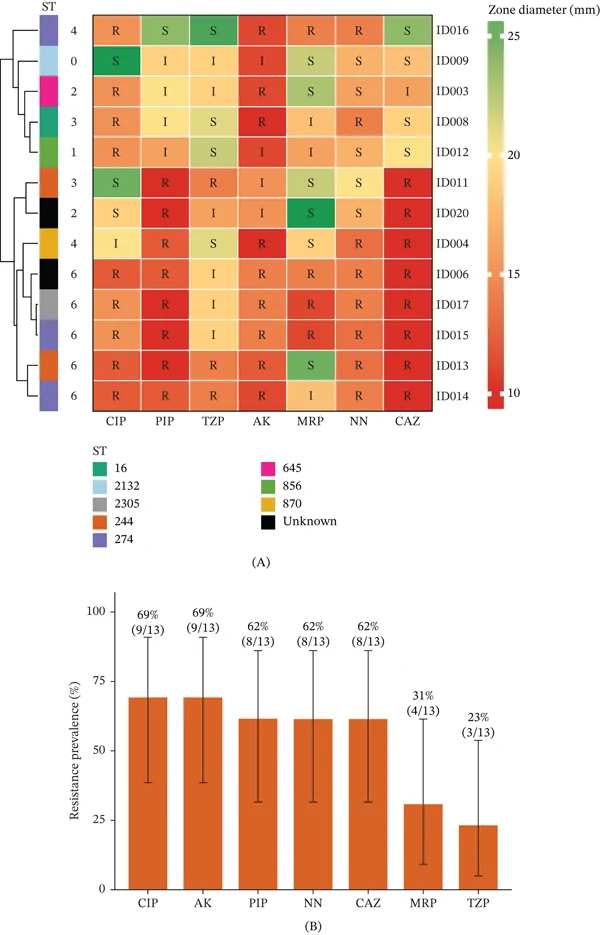
Antimicrobial susceptibility profiles of the sequenced isolates. (A) Hierarchically clustered heatmap based on inhibition zone diameters obtained by disk diffusion analysis. Cell colors represent diameters of inhibition zone (mm), whereas letters within cells indicate CLSI susceptibility classifications (S, susceptible; I, intermediate; R, resistant). Annotations indicate multilocus sequence type (ST) and multidrug resistance (MDR) score, defined in this study as the number of antibiotics for which an isolate was classified as resistant. Hierarchical clustering of susceptibility profiles was performed using Euclidean distance matrices derived from inhibition zone diameters. (B) Resistance prevalence for the seven antibiotics tested. Bars represent the percentage of isolates classified as resistant according to CLSI 2017 interpretive criteria. Error bars indicate exact 95% binomial confidence intervals. Values above bars denote the percentage of resistant isolates, with the corresponding number of resistant isolates relative to the total number tested shown in parentheses. CIP, ciprofloxacin; PIP, piperacillin; TZP, piperacillin/tazobactam; AK, amikacin; MRP, meropenem; NN, tobramycin; CAZ, ceftazidime.

Antimicrobial susceptibility profiles varied substantially among the isolates (Figure 1A). Hierarchical clustering based on inhibition zone diameters identified a group of highly resistant isolates (ID006, ID013–ID015, and ID017), characterized by elevated MDR scores and resistance to multiple antibiotic classes. In contrast, isolates such as ID009, ID011, and ID020 showed broader susceptibility profiles. Across the collection, resistance was most frequently observed against AK, CAZ, and PIP, whereas MRP retained activity against a larger proportion of isolates (Figure 1B and Data S1).

**Figure 2:**
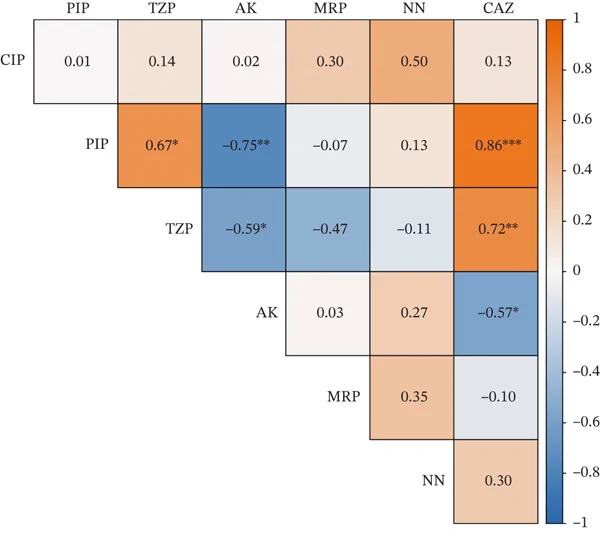
Spearman correlation matrix of inhibition zone diameters obtained for the seven antibiotics tested. Correlation coefficients (*ρ*) are shown within cells and color intensity reflects the strength and direction of the association, ranging from negative (blue) through no correlation (white) to positive (orange). Asterisks denote statistically significant correlations (∗*p* < 0.05, ∗∗*p* < 0.01, ∗∗∗*p* < 0.001).

Correlation analysis identified several significant associations between antibiotic susceptibility profiles (Figure 2). The strongest positive correlation was observed between PIP and CAZ (Spearman^′^s *ρ* = 0.86, *p* < 0.001), followed by TZP and CAZ (*ρ* = 0.72, *p* = 0.005) and PIP and TZP (*ρ* = 0.67, *p* = 0.012). Significant negative correlations were observed between PIP and AK (*ρ* = −0.75, *p* = 0.003), TZP and AK (*ρ* = −0.59, *p* = 0.035), and AK and CAZ (*ρ* = −0.57, *p* = 0.042). No statistically significant correlations were detected among the remaining antibiotic pairs (Data S2).

**Figure 3:**
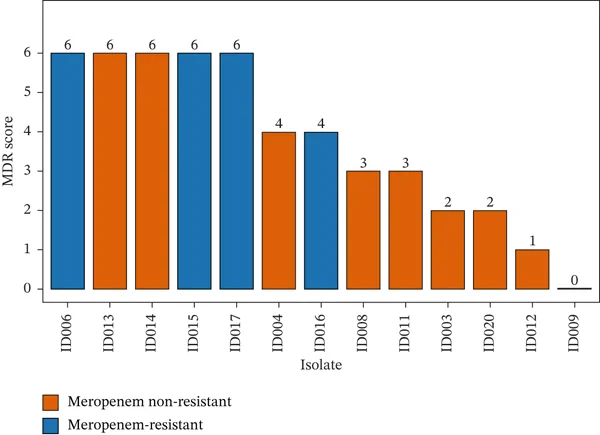
Multidrug resistance (MDR) burden across the *P. aeruginosa* isolates. MDR burden was calculated as the number of antibiotics for which each isolate was classified as resistant according to CLSI criteria. Bars are colored according to meropenem susceptibility status.

In this study, the strains showing resistance to MRP were also characterized by a rather high level of overall resistance against all other antibiotics. Since the MDR score is the number of antibiotics classified as resistant, we wanted to understand if MRP is associated with higher MDR burden via Wilcoxon *t*‐test. Here, MDR burden varied across the isolate collection, with MDR scores ranging from 0 to 6 resistant phenotypes per isolate (Figure 3). Five isolates had the highest MDR burden (MDR score = 6), whereas one isolate showed no resistance to the antibiotics tested. MRP‐resistant isolates generally had higher MDR scores than MRP‐nonresistant isolates (median MDR score: 6 vs. 3), although this difference did not reach statistical significance (Wilcoxon rank‐sum test, *p* = 0.056) (Data S3).

### 3.2. Sequencing and Annotation

**Figure 4:**
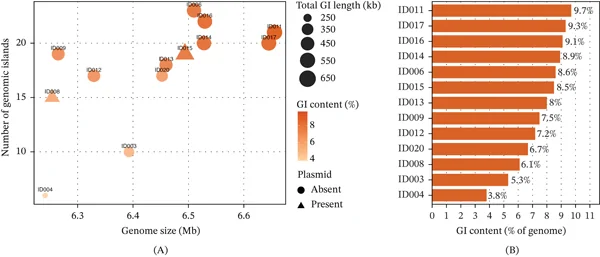
Genome architecture and genomic island content of the *Pseudomonas aeruginosa* isolates. (A) Relationship between chromosome size and the abundance of genomic islands. Bubble size corresponds to the cumulative length of genomic islands, and color intensity is the proportion of the chromosome occupied by genomic islands. Symbols indicate plasmid carriage. (B) Genomic island content expressed as the percentage of chromosome length occupied by predicted genomic islands, with isolates ranked in descending order.

In this study, genome sizes of the isolates ranged from 6.24 Mb (ID004) to 6.66 Mb (ID011), with the number of chromosomal coding sequences (CDSs) varying from 5672 to 6061 (Figure 4A). Plasmids were detected in only two isolates: ID008 carried a 46,344‐bp plasmid containing 48 CDSs, whereas ID015 harbored a 2259‐bp plasmid encoding 3 CDSs. Predicted GIs were identified in all genomes, with GI counts ranging from 6 (ID004) to 23 (ID006). The cumulative length of GIs varied from 242,666 to 650,092 bp, corresponding to 247–585 GI‐associated CDSs per genome. GIs accounted for between 3.8% and 9.7% of chromosome length across the isolate collection (Figure 4B). A complete summary of genome assembly and annotation metrics is provided in Data S4_1.

**Figure 5:**
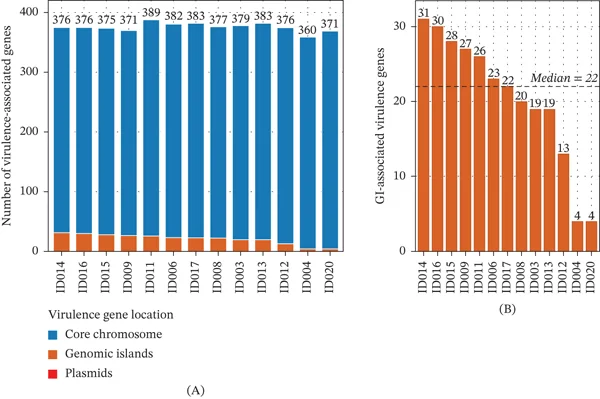
Distribution of virulence‐associated genes among the sequenced PA isolates. (A) Partitioning of virulence‐associated genes between the core chromosome, genomic islands (GIs), and plasmids. Stacked bars represent the total number of virulence‐associated genes detected in each isolate, with colors indicating their genomic location. Values above bars denote the total virulence gene count per isolate. Core chromosomal virulence genes were largely conserved across genomes, whereas the contribution of GI‐associated virulence genes varied among isolates. Plasmid‐associated virulence genes were detected only in isolate ID008. (B) Ranked abundance of GI‐associated virulence genes across the isolate collection. Isolates are ordered from highest to lowest GI‐associated virulence gene content. Values above bars indicate the number of virulence‐associated genes located within genomic islands. The dashed horizontal line represents the median GI‐associated virulence gene count across all isolates. Detailed distributions of individual virulence determinants among genomic islands are provided in Data S4_2.

In this study, virulence‐associated genes were partitioned between the conserved core chromosome and the accessory genome, including GIs and plasmids (Figure 5A). Core chromosomal virulence gene content was highly conserved across isolates, ranging from 344 to 367 genes. On the other hand, GI‐associated virulence genes showed substantial variation, ranging from 4 genes in isolates ID004 and ID020 to 31 genes in isolate ID014 (Figure 5B). Isolates ID014 and ID016 carried the largest GI‐associated virulence repertoires, harboring 31 and 30 GI‐associated virulence genes, respectively, whereas isolates ID004 and ID020 contained the fewest. Plasmid‐associated virulence genes were detected only in isolate ID008, which encoded two such genes. Overall, variation in total virulence gene content among isolates was primarily attributable to differences in the accessory virulence repertoire associated with GIs rather than the conserved core chromosome.

### 3.3. Genome Comparison

**Figure 6:**
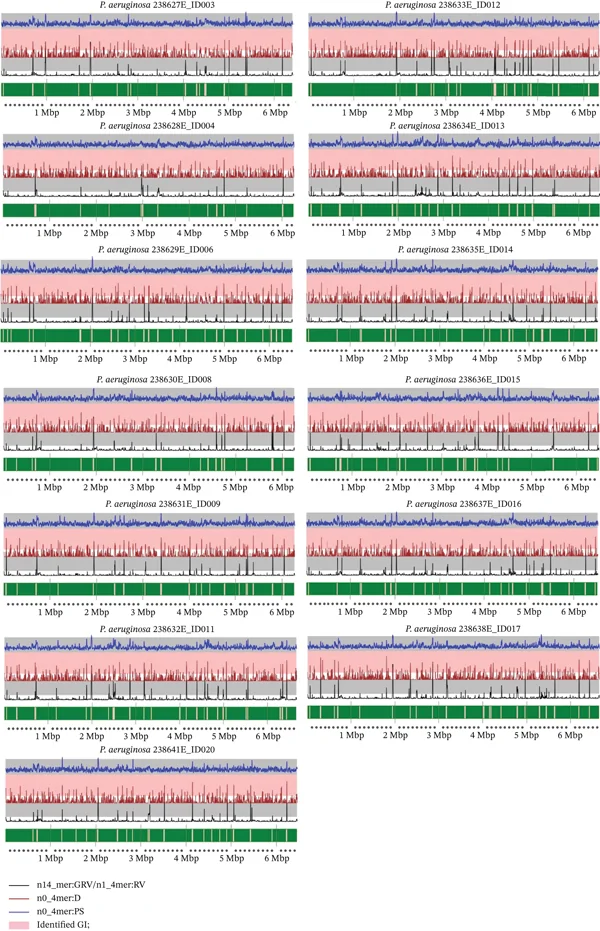
Distribution of genomic islands (GIs) across the chromosomes of the sequenced PA isolates. GIs were identified using SeqWord Gene Island Sniffer based on local deviations in oligonucleotide composition relative to the genomic background. For each genome, the upper tracks show genome‐wide variation in tetranucleotide (4‐mer) composition, including the generalized relative variance (GRV/RV) ratio [23]. It also shows the tetranucleotide pattern skew (PS) and the distance between local and genome‐wide 4‐mer frequency distributions. Regions highlighted in pink represent predicted GIs, whereas the lower green track shows the chromosomal gene map. Predicted GIs correspond to regions exhibiting atypical nucleotide composition relative to the remainder of the chromosome, consistent with acquisition through horizontal gene transfer.

Several GIs were identified across the sequenced isolates. Multiple GIs showed deviations in local 4‐mer composition relative to the overall genomic background. For example, a major GI in isolate ID011 showed a distinct 4‐mer profile compared with the remainder of the genome (Figure 6). Similar differences in local versus genome‐wide nucleotide composition were observed in a GI of isolate ID020. Isolate ID009 contained several GIs characterized by irregular 4‐mer patterns and disrupted gene organization. In contrast, isolate ID013 had fewer compositional anomalies, with only one GI displaying a marked deviation in 4‐mer composition. Transposase‐encoding genes were detected in several GIs. For example, the Tn3 family transposase Tn5393 was identified in GI#6 of ID017. Isolate ID006 encoded the IS4 family transposase ISPen1 in GI#1, GI#4, GI#7, GI#9, and GI#16. Isolate ID020 contained two transposases, the IS3 family transposase ISPa86 and a REP‐associated tyrosine transposase, located in GI#6 and GI#13, respectively (Data S4_2). The IS3 family transposase ISPa32 was identified in isolates ID011, ID013, ID014, ID015, and ID016.

### 3.4. Phylogenetic Relationships Between *P. aeruginosa* Strains

**Figure 7:**
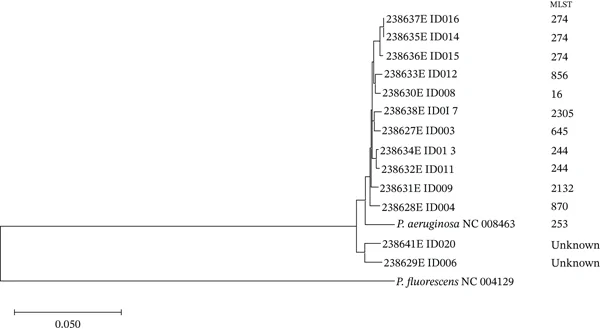
Neighbor‐joining phylogenetic tree showing the evolutionary relationships among the PA isolates based on a concatenated amino acid alignment of 2978 orthologous protein‐coding genes identified using OrthoFinder. Individual orthologous protein sequences were aligned with MUSCLE and concatenated into a superalignment comprising 975,355 amino acid positions. Phylogenetic reconstruction was performed using the JTT amino acid substitution model. Multilocus sequence types (MLSTs) are indicated alongside the corresponding isolates. The genome of *Pseudomonas fluorescens* NC_004129 was included as an outgroup for tree rooting. Branch lengths are proportional to evolutionary divergence, and the scale bar represents the estimated number of amino acid substitutions per site.

The phylogenetic reconstruction revealed substantial genomic diversity among the isolates (Figure 7). Three isolates (ID014, ID015, and ID016) formed a well‐supported cluster and were assigned to ST274. Isolates ID012 (ST856) and ID008 (ST16) also grouped closely, despite belonging to different sequence types. The remaining isolates were distributed across several distinct lineages, including ST645 (ID003), ST244 (ID011 and ID013), ST870 (ID004), ST2132 (ID009), and ST2305 (ID017). The reference strain PAO1 (ST253) clustered within the main *P. aeruginosa* clade but remained distinct from the study isolates. Notably, isolates ID006 and ID020 formed a separate branch within the *P. aeruginosa* lineage and could not be assigned a known sequence type using the PubMLST database and had previously uncharacterized allelic profiles.

### 3.5. Drug Resistance Genes and Virulence Determinants

**Figure 8:**
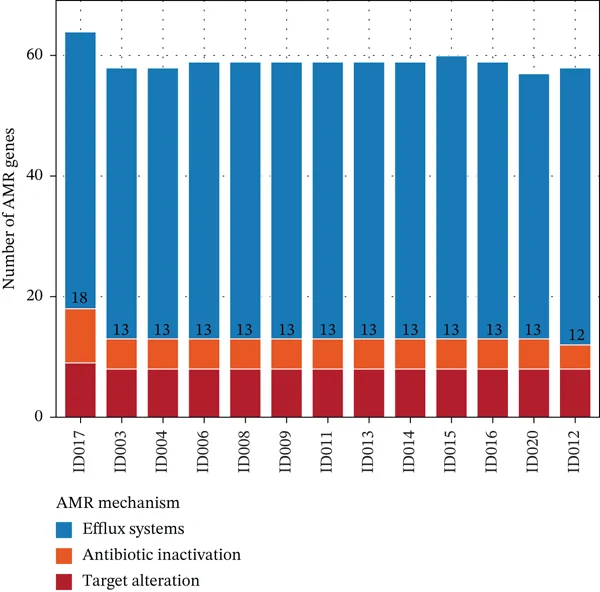
Distribution of antimicrobial resistance (AMR) genes by mechanism of action across PA isolates. Stacked bars represent the abundance of AMR genes assigned to three major resistance mechanisms: efflux systems, antibiotic inactivation, and target alteration. Values above bars indicate the total number of AMR genes identified in each isolate. Efflux‐associated determinants constituted the largest resistance mechanism category in all genomes, whereas genes involved in antibiotic inactivation and target alteration were less abundant. Isolates are ordered according to accessory AMR gene content (antibiotic inactivation and target alteration genes).

A total of 54 AMR genes and 240 virulence‐associated genes were identified across the sequenced genomes (Data S5 and S6). AMR genes identified across the genomes were predominantly associated with efflux systems, which represented the largest resistance mechanism category in all isolates (Figure 8). Efflux‐associated genes ranged from 44 to 47 per genome, whereas genes involved in antibiotic inactivation and target alteration were less abundant, ranging from 4 to 9 and 8–9 genes, respectively. Overall, resistance mechanism profiles were highly conserved among isolates, although isolate ID017 carried the highest numbers of antibiotic inactivation and target alteration genes (Figure 8 and Data S4_3). This pattern indicates that multidrug efflux systems constitute the principal genomic resistance strategy within the isolate collection.

**Figure 9:**
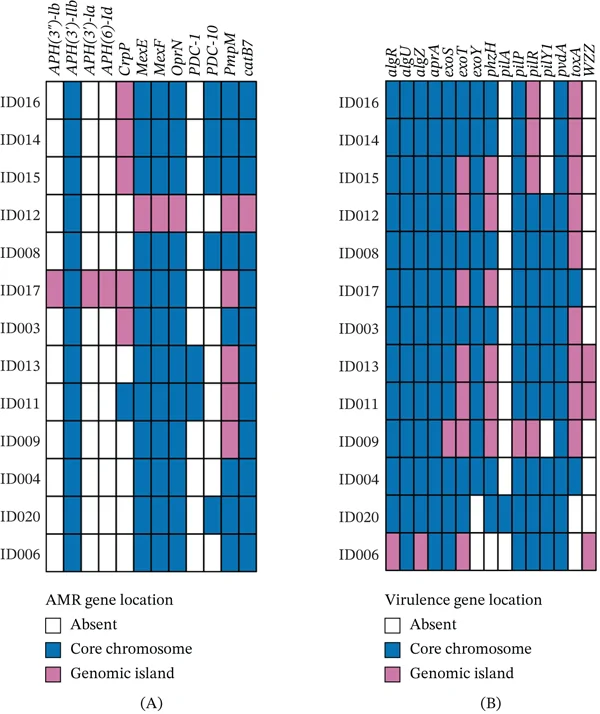
Distribution and genomic localization of selected antimicrobial resistance and virulence‐associated genes among sequenced PA isolates. (A) The presence and genomic location of selected antimicrobial resistance determinants. Rows represent isolates ordered according to the core‐genome phylogeny (Figure 7), and columns represent selected resistance genes. White cells indicate gene absence, blue cells indicate genes located within the chromosomal core genome, and purple cells indicate genes located within genomic islands (GIs). Genes were selected based on their genomic variability, GI association, or clinical relevance and include aminoglycoside resistance genes (APH (3 ^′^)‐Ia, APH (6)‐Id, and APH (3 ^″^)‐Ib), the fluoroquinolone resistance gene crpP, multidrug efflux determinants (mexE, mexF, and oprN), *β*‐lactamase genes (PDC‐1 and PDC‐10), the phenicol resistance gene catB7, and the multidrug efflux transporter pmpM. (B) The distribution and genomic localization of selected virulence‐associated genes showing their occurrence and their association with GIs. Rows represent isolates ordered according to the core‐genome phylogeny (Figure 7), and columns represent selected virulence genes involved in alginate regulation, Type III secretion, pilus biogenesis, iron acquisition, toxin production, phenazine biosynthesis, and lipopolysaccharide regulation. White, orange, and red cells indicate gene absence, localization within the chromosomal core genome, and localization within genomic islands, respectively. Complete AMR and virulence gene inventories and genomic locations are provided in Data S5 and S6.

Selected AMR determinants showed variable distributions and genomic locations across the sequenced *P. aeruginosa* isolates (Figure 9A and Table S5). The aminoglycoside resistance gene APH (3 ^′^)‐IIb was detected in all isolates and was consistently located within the chromosomal core genome. In contrast, APH (3 ^′^)‐Ia, APH (6)‐Id, and APH (3 ^″^)‐Ib were detected exclusively in isolate ID017 and were localized within GI#6. The fluoroquinolone resistance gene *CrpP* was identified in several isolates and was associated with GIs, although the specific GI differed among strains. The multidrug efflux genes MexE, MexF, and OprN were located within GI#14 of isolate ID012, whereas the phenicol resistance gene catB7 was located within GI#15 of the same isolate. The polymyxin resistance‐associated regulator *β*‐lactamase genes PDC‐1 and PDC‐10 showed variable distributions across the isolate collection and were present either in the chromosomal core genome or within GIs depending on the strain. Likewise, the multidrug efflux transporter PmpM was broadly distributed among isolates but was associated with GIs in several strains. Overall, these findings demonstrate that the AMR repertoire comprises both conserved chromosomal determinants and strain‐specific GI‐associated resistance genes. Complete AMR gene inventories and genomic locations are provided in Table S5.

Virulence‐associated genes also varied in genomic distribution among isolates (Figure 9B and Table S6). Although many virulence determinants formed part of the conserved chromosomal core genome, several genes were associated with GIs and showed strain‐specific distributions. Notable examples included *algR* and *algZ* within GI#22 of isolate ID006 and *exoS* within GI#3 of isolate ID009. Additional variation was observed among genes involved in type III secretion, pilus biogenesis, phenazine biosynthesis, and lipopolysaccharide regulation, several of which occurred within GIs in specific isolates. Overall, the observed distribution patterns indicate that both conserved and accessory virulence determinants contribute to genomic diversity within the isolate collection.

### 3.6. QS in *P. aeruginosa* Isolates

**Figure 10:**
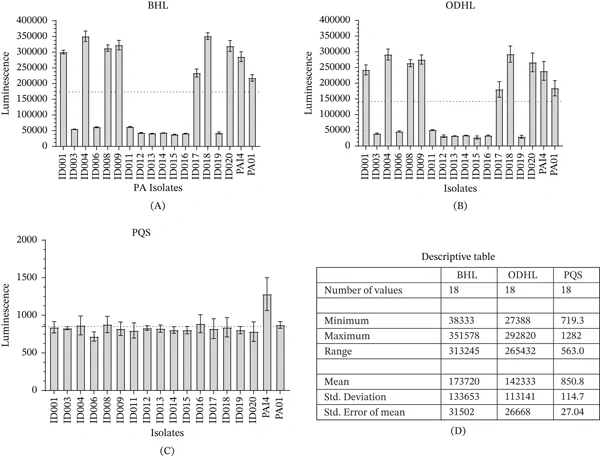
Quorum‐sensing signal production by PA isolates. (A) N‐butanoyl‐L‐homoserine lactone (BHL), (B) N‐(3‐oxododecanoyl)‐L‐homoserine lactone (OdDHL), and (C) Pseudomonas quinolone signal (PQS) production were measured using luminescent biosensor strains. Bars represent mean luminescence values obtained from triplicate assays, and error bars indicate standard deviations. Dotted horizontal lines indicate the luminescence response of the reference strain *P. aeruginosa* PAO1, and are shown as a comparative benchmark for signal production. Isolates with luminescence values above the reference threshold exhibited elevated production of the corresponding quorum‐sensing signal. (D) Descriptive statistics summarized the distribution of luminescence values for each signal across all isolates, including minimum, maximum, range, mean, standard deviation, and standard error of the mean. Luminescence was measured using a Lucy 1 single‐point luminometer (Anthos Labtec Instruments, Austria). BHL, N‐butanoyl‐L‐homoserine lactone; OdDHL, N‐(3‐oxododecanoyl)‐L‐homoserine lactone; PQS, Pseudomonas quinolone signal.

**Figure 11:**
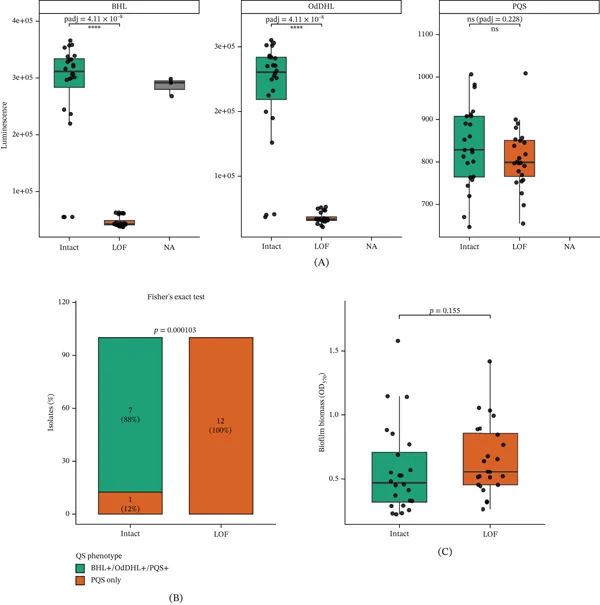
Association between quorum‐sensing (QS) regulator genotype, quorum‐sensing phenotype, and biofilm formation in PA clinical isolates. (A) Comparison of QS signal production according to QS regulator genotype. Boxplots show luminescence values measured for N‐butanoyl‐L‐homoserine lactone (BHL), N‐(3‐oxododecanoyl)‐L‐homoserine lactone (OdDHL), and the Pseudomonas quinolone signal (PQS) biosensor assays following classification of isolates into Intact (lasR and rhlR intact) and LOF (predicted loss‐of‐function mutations in lasR and/or rhlR) groups. Individual points represent technical triplicates. Boxes indicate the interquartile range, center lines represent the median, and whiskers extend to 1.5x the interquartile range. Differences between groups were assessed using the Wilcoxon rank‐sum test with Benjamini–Hochberg correction for multiple comparisons. Adjusted *p* values are shown above each panel. (B) Distribution of QS phenotypes among isolates with intact or loss‐of‐function QS regulators. Bars show the percentage of isolates showing either complete QS activity (BHL+/OdDHL+/PQS+) or a PQS‐only phenotype within each genotype group. Numbers within bars indicate isolate counts and percentages. The association between QS regulator genotype and QS phenotype was evaluated using Fisher′s exact test, with the resulting *p* value shown above the bars. (C) Comparison of biofilm biomass between isolates with intact QS regulators and those carrying predicted loss‐of‐function mutations in lasR and/or rhlR. Biofilm formation was quantified by the crystal violet microtiter plate assay and expressed as optical density at 570 nm (OD<sub>570</sub>). Individual points represent technical triplicates. Boxes indicate the interquartile range, center lines represent the median, and whiskers extend to 1.5x the interquartile range. Differences between genotype groups were assessed using the Wilcoxon rank‐sum test, and the corresponding *p* value is shown above the plot.

Production of the QS signal molecules BHL, OdDHL, and PQS varied among the *P. aeruginosa* isolates (Figure 10). The greatest variation was observed for the acyl‐homoserine lactone (AHL) signals BHL and OdDHL, which had a clear bimodal distribution, with several isolates producing high signal levels, whereas others produced little or no detectable signal. In contrast, production of PQS was detected in all isolates and showed comparatively limited variation across the collection. Isolates producing high levels of BHL generally also produced elevated levels of OdDHL, consistent with coordinated activity of the AHL‐dependent QS systems. Representative high producers included isolates ID001, ID004, ID008, ID009, ID017, ID018, and ID020, whereas the remaining isolates showed reduced or undetectable production of one or both AHL signals. Comparison of QS signal production according to QS regulator genotype showed significant differences for the two AHL signals (Figure 11A). Isolates with intact *lasR* and *rhlR* produced significantly higher levels of both BHL and OdDHL than isolates carrying predicted LOF mutations in one or both regulators (Wilcoxon rank‐sum test, Benjamini–Hochberg adjusted *p* = 4.11 × 10^−8^ for both comparisons). In contrast, PQS production did not differ significantly between the two genotype groups (adjusted *p* = 0.228). Summary statistics, effect sizes, and complete statistical results are provided in Table S7.

Further comparison of QS regulator genotype with experimentally determined QS phenotype demonstrated a significant association between predicted regulator function and signal production (Figure 11B). All isolates carrying predicted LOF mutations in *lasR* and/or *rhlR* showed a PQS‐only phenotype, whereas 87.5% (7/8) of isolates with intact QS regulators produced all three QS signal molecules (BHL, OdDHL, and PQS). Only one isolate with intact QS regulators showed a PQS‐only phenotype. Fisher′s exact test confirmed a significant association between QS regulator genotype and QS phenotype (*p* = 0.000103).

To determine whether QS regulator genotype was associated with biofilm formation, crystal violet biofilm biomass was compared between genotype groups (Figure 11C). Although isolates with predicted LOF mutations exhibited a slightly higher median biofilm biomass than isolates with intact QS regulators, the distributions overlapped substantially and the difference was not statistically significant (Wilcoxon rank‐sum test, *p* = 0.155).

Finally, associations between accessory AMR determinants and QS regulator genotype were evaluated using Fisher′s exact tests. Although QS regulator genotype was strongly associated with AHL production and overall QS phenotype, no significant association was observed with biofilm biomass or accessory AMR gene carriage after Benjamini–Hochberg correction (File S8 and Figure S1).

## 4. Discussion

We sought to characterize the genomic diversity, AMR, virulence potential, and QS phenotypes of *P*. *aeruginosa* isolates recovered from SSIs in Western Kenya. Together with previous genomic investigation of *Staphylococcus aureus* from the same clinical setting [26], these findings demonstrate that surgical site pathogens circulating within this hospital exhibit substantial genomic diversity and evidence of ongoing genome evolution. By integrating antimicrobial susceptibility tests, whole‐genome sequencing, comparative genomics, and QS analyses, we demonstrate substantial genomic diversity despite a relatively small collection of clinical isolates. Although MDR was common, carbapenem susceptibility remained largely preserved, and genomic analyses revealed considerable variation in mobile genetic elements, accessory AMR determinants, virulence‐associated genes, and QS phenotypes. Collectively, these findings highlight the dynamic evolution of *P. aeruginosa* within the local hospital environment [27]. They further underscore the importance of combining genomic and phenotypic methodologies for surveillance.

In this study, the antimicrobial susceptibility profiles were consistent with the intrinsic resistance characteristics of *P. aeruginosa* and with increasing MDR reported globally [28, 29]. Resistance was most frequently observed against fluoroquinolones and several *β*‐lactams, whereas MRP and TZP retained the greatest in vitro activity. These findings suggest that carbapenems remain effective therapeutic options in this setting, although their continued efficacy depends on appropriate antimicrobial stewardship. This is so because increasing resistance to carbapenem has been documented in PA and other clinically important nosocomial pathogens [30–32]. Our genomic analyses support these phenotypic observations by showing that intrinsic resistance determinants, including efflux‐associated genes and chromosomal *β*‐lactamases [33], were widely conserved. This is consistent with the central contribution of these mechanisms to multidrug resistance in clinically important gram‐negative pathogens [6, 34]. More broadly, regulatory mutations have also been shown to simultaneously influence AMR and virulence in *P. aeruginosa*, highlighting the interconnected nature of these adaptive traits [35]. We also noted that aminoglycoside‐modifying enzymes [36], and the fluoroquinolone resistance gene *crpP* occurred only in a small number of isolates and were associated with GIs. This is consistent with sporadic horizontal acquisition of these genes rather than dissemination [37].

Further, comparative genomic analyses revealed substantial diversity in GIs, insertion sequences, and virulence‐associated genes across our isolate collection. Similar to previous reports that described the contribution of GIs to the evolution of high‐risk *P. aeruginosa* lineages [9, 38], several isolates contained large GIs harboring resistance‐ and virulence‐associated genes. However, these accessory elements were unevenly distributed among isolates, emphasizing that horizontal gene transfer [39], continues to shape genome evolution even within a single healthcare facility [8]. A similar pattern of genomic hotspots associated with pathogen evolution was also observed among *S. aureus* surgical site isolates recovered from the same hospital [26], suggesting that genome plasticity is a common feature of clinically important SSI pathogens in this setting. Although certain virulence determinants were localized within GIs, the majority of genes examined remained part of the conserved chromosomal backbone. This is indicative of both core and accessory genomes contributing to the pathogenic potential of clinical *P. aeruginosa.* Additionally, the identification of plasmids in two isolates, despite the absence of additional resistance or virulence determinants, further underscores the potential for future acquisition of clinically relevant genes through horizontal gene transfer [40].

A major finding of this study was the strong relationship between QS regulator genotype and QS phenotype. Isolates with intact *lasR* and *rhlR* produced significantly greater quantities of the acyl‐homoserine lactone signals BHL and OdDHL than isolates carrying predicted LOF mutations, whereas PQS production remained comparatively conserved. This observation is consistent with the hierarchical organization of the *P. aeruginosa* QS network. Here, e*LasR* and *RhlR* primarily regulate acyl‐homoserine lactone signaling, whereas PQS can remain active through partially independent regulatory pathways [10, 41]. The significant association between QS regulator genotype and experimentally determined QS phenotype further supports the functional importance of *lasR* and *rhlR* integrity in maintaining coordinated QS activity. In contrast, biofilm biomass did not differ significantly between QS genotype groups, suggesting that biofilm formation under the experimental conditions is rather influenced by additional regulatory pathways beyond the canonical QS network. Likewise, the carriage of the accessory AMR determinants examined was not associated with QS regulator genotype, indicating that the acquisition of these mobile resistance genes occurred independently of QS regulatory status within this isolate collection.

Several limitations should be considered when interpreting these findings. The study represents isolates collected from a single referral hospital and therefore may not fully capture the genomic diversity of *P. aeruginosa* circulating elsewhere in Kenya. In addition, the relatively small number of sequenced isolates limits statistical power for detecting associations involving infrequently distributed accessory genes. Nevertheless, our work provides a detailed overview of the diversity of clinically relevant *P. aeruginosa* in this setting. Continued genomic surveillance incorporating larger isolate collections and longitudinal sampling will be important for monitoring the emergence and dissemination of high‐risk lineages [42, 43]. This also includes mobile resistance determinants and alterations in QS‐associated virulence.

## 5. Conclusion

This study provides a comprehensive genomic and phenotypic characterization of *P*. *aeruginosa* isolates recovered from SSIs in Western Kenya. Although MDR was common, MRP and TZP retained the greatest in vitro activity. Whole‐genome analyses revealed great diversity in sequence types, GIs, virulence‐associated genes, and accessory AMR determinants. This is yet another significant highlight of the important contribution of horizontal gene transfer to the pathogen′s evolution. Quantitative analyses further demonstrated that intact *lasR* and *rhlR* regulators were strongly associated with acyl‐homoserine lactone QS activity and overall QS phenotype, whereas biofilm formation and carriage of accessory AMR determinants were not associated with QS regulator genotype. Our findings emphasize that although QS regulator integrity influences signaling phenotypes, acquisition of accessory resistance genes appears to occur independently of QS status in this isolate collection.

Together, these findings establish an important genomic baseline for *P. aeruginosa* causing SSIs in Western Kenya and underscore the value of integrating antimicrobial susceptibility testing, whole‐genome sequencing, and phenotypic characterization for surveillance. Continued genomic surveillance, coupled with antimicrobial stewardship, will be essential to monitor not only the emergence and dissemination of clinically important PA lineages, but also mobile genetic elements within healthcare settings.

## Author Contributions

Sarah Kindiki, Sabella Kiprono, and Anthony Sifuna were involved in the conceptualization of the study, the methodology, and the writing of the original manuscript draft. Oleg Reva. and Nyabera Nicholas Mogoi were involved in data curation, bioinformatics, manuscript writing, and correction. Nyabera Nicholas Mogoi was involved in statistical analyses and graphical visualizations. Sarah Kindiki and Peter Kuloba Nyongesa were involved in the sample collection and laboratory procedures. Martin Welch was involved in reviewing and editing the manuscript. Anthony Sifuna, Oleg Reva, and Martin Welch acquired the funding.

## Funding

This study was supported by the Cambridge‐Africa ALBORADA Research fund (G115009).

## Conflicts of Interest

The authors declare no conflicts of interest.

## Supporting information


**Supporting Information** Additional supporting information can be found online in the Supporting Information section. Table S1 presents the antimicrobial susceptibility profiles and related characteristics of the *Pseudomonas aeruginosa* isolates included in the study. Data S2 presents the correlation analysis of antimicrobial susceptibility measurements among the tested antibiotics. Data S3 presents the multidrug resistance (MDR) burden and comparison of MDR profiles according to meropenem resistance status. Data S4_1 provides detailed genome assembly and annotation metrics for the sequenced isolates, whereas Data S4_2 provides detailed distributions of genomic island–associated virulence determinants and insertion sequences. Data S4_3 provides additional information on antimicrobial resistance mechanisms and accessory antimicrobial resistance gene content. Table S5 presents the complete inventory and genomic locations of antimicrobial resistance genes identified in the sequenced isolates. Table S6 presents the complete inventory and genomic locations of virulence‐associated genes. Table S7 provides summary statistics, effect sizes, and statistical results for the quorum‐sensing signal production analyses. File S8 provides the statistical analysis of associations between quorum‐sensing regulator genotype, biofilm biomass, and accessory antimicrobial resistance gene carriage. Figure S1 presents additional graphical results of these analyses. File S9 contains the study consent form and data collection questionnaire.

## Data Availability

The data that support the findings of this study are available from the corresponding author upon reasonable request. The R scripts used for statistical analyses and figure generation are publicly available at the authors′ GitHub repository: https://github.com/N-mogoi/genomic_analyses_PA_WKenya.git. The repository contains the scripts required to reproduce the analyses and figures presented in this study.
